# The Good, the Bad, and the Clever: Faking Ability as a Socio-Emotional Ability?

**DOI:** 10.3390/jintelligence9010013

**Published:** 2021-03-04

**Authors:** Mattis Geiger, Romy Bärwaldt, Oliver Wilhelm

**Affiliations:** 1 Institute of Psychology and Education, Ulm University, 89069 Ulm, Germany; oliver.wilhelm@uni-ulm.de; 2 Department of Psychology, University of Münster, D-48149 Münster, Germany; romy.baerwaldt@uni-muenster.de

**Keywords:** faking good, faking bad, faking ability, socio-emotional abilities, productive and receptive abilities, general mental abilities

## Abstract

Socio-emotional abilities have been proposed as an extension to models of intelligence, but earlier measurement approaches have either not fulfilled criteria of ability measurement or have covered only predominantly receptive abilities. We argue that faking ability—the ability to adjust responses on questionnaires to present oneself in a desired manner—is a socio-emotional ability that can broaden our understanding of these abilities and intelligence in general. To test this theory, we developed new instruments to measure the ability to fake bad (malingering) and administered them jointly with established tests of faking good ability in a general sample of *n* = 134. Participants also completed multiple tests of emotion perception along with tests of emotion expression posing, pain expression regulation, and working memory capacity. We found that individual differences in faking ability tests are best explained by a general factor that had a large correlation with receptive socio-emotional abilities and had a zero to medium-sized correlation with different productive socio-emotional abilities. All correlations were still small after controlling these effects for shared variance with general mental ability as indicated by tests of working memory capacity. We conclude that faking ability is indeed correlated meaningfully with other socio-emotional abilities and discuss the implications for intelligence research and applied ability assessment.

## 1. Introduction

Because social interaction is such an integral part of human life, psychological research has introduced a virtually endless list of socio-emotional constructs. This research suffers from challenges in assessment that resulted in a proliferation of constructs, leading to jingle and jangle fallacies (Olderbak and Wilhelm 2020). However, studies on some of these constructs, namely socio-emotional abilities (with the term socio-emotional abilities, we refer to abilities related to processing, producing, and regulating social and emotional information), have demonstrated their importance in expanding models of intelligence (Hildebrandt et al. 2011, 2015; MacCann et al. 2014; Olderbak et al. 2019a; Schlegel et al. 2019, 2020; Schlegel and Scherer 2018) and predicting real-life outcomes (Côté et al. 2010; Joseph and Newman 2010; MacCann et al. 2020). Still, most research in this field has focused on basic socio-emotional abilities, such as emotion perception and recognition.

With this paper, we strive to expand the construct of socio-emotional abilities to more complex abilities that might be more ecologically valid. The ability to deceive is one such complex socio-emotional ability (Riggio et al. 1987). Research on successful deception detection tells us what is necessary for successful deception (Bond and DePaulo 2006): deceivers must align their behavior to the situational context they are in. To do so, they must understand the situational demands and produce matching behavior (Vrij 2002). For example, if a person wants to create a deception about getting to know another person for the first time, the deceiver should understand that it is polite and a custom to introduce themselves on a first meeting and behave accordingly. Furthermore, the deceiver should react surprised, e.g., via facial expressions, when they learn something surprising about the other person, even if they already knew it. In sum, next to knowledge about situational demands, deception requires receptive and productive socio-emotional abilities to be successful.

Studying deception with such complex interpersonal interactions, such as the example above, illustrates the issue of sender-receiver duality in judging the success of deception. Typical approaches to measuring deception ability have a deceiver act out lies (and truths) and then have the lies (and truths) classified by an independent group of judges (Bond and DePaulo 2008). For example, deceivers tell emotional or unemotional stories about their life (e.g., Law et al. 2018) or they roll a die and can decide to tell the truth or to lie (e.g., a Meyer game as described in Sip et al. 2010) and judges see them either live or videos of lies/truths and then classify whether what they saw was supposedly true or a lie. Thus, any deception ability scores from such tests hinge upon the judges and their deception detection ability. Obviously, this undermines any such deception test’s objectivity and limits the comparability of studies using different judges.

### 1.1. Faking

To solve this issue, we can study deception behavior where the respective goal can be operationalized in an objective manner. One prominent example of this is faking, a specific instance of deception behavior (Melchers et al. 2020b). Faking refers to an intentional distortion of responses in assessments in order to achieve a certain goal such as getting a job or a diagnosis. In other words, fakers respond with deceiving answers instead of providing their “true” answers. Faking is a frequent phenomenon and common issue in psychological testing (Hall and Hall 2011). For example, in job selection contexts, participants tend to answer in a way that makes them appear more conscientious and emotionally stable than they actually are (Birkeland et al. 2006; Viswesvaran and Ones 1999). Similarly, in clinical assessment, malingering—faking symptoms—is prevalent (Hall and Hall 2011).

As these examples show, faking behavior can be distinguished as faking good and faking bad. Faking good refers to an attempt to appear better than is actually the case; faking bad refers to attempts to appear worse than actually is the case. Participants instructed to fake easily grasp this distinction and fake accordingly (Bensch et al. 2019): faking good and bad are understood as different situational demands. Situational demands can also differ within faking good (Geiger et al. 2018; Pelt et al. 2018) or bad conditions, i.e., faking good for different jobs has different situational demands.

The goal of faking is to meet these situational demands. Whether the goal of faking is reached, i.e., faking performance, is determined by three factors (Tett and Simonet 2011): opportunity, motivation, and ability. Motivation and opportunity can often be considered at a maximum in real-world faking settings, leaving faking ability as the driving factor of performance (Geiger et al. 2018). Whether faking performance is determined by a general faking ability or independent ability factors based on situational demands (e.g., faking good vs. faking bad) is an empirical question that we will address in this manuscript. Considering not only the distinct response patterns that arise when faking good versus bad (Bensch et al. 2019), but also that the typical situations in which they occur differ, it might be argued that faking good and bad form distinct factors. However, the ability to fake good for highly distinct jobs is correlated between these conditions, although they have different situational demands (Pelt et al. 2018) and is best described as a common ability factor (Geiger et al. 2018).

### 1.2. Assessing Faking Ability

The assessment of faking ability depends on the instrument that is faked. Among fakeable measurement approaches in psychological assessment, interviews and questionnaires are dominant. Faking in interviews is studied under the umbrella term ability to identify criteria (ATIC; Kleinmann et al. 2011; König et al. 2006), a prerequisite to faking behavior in interviews. ATIC refers to participants’ skills to identify traits targeted by interview questions. For example, a question about theoretical conflict between the interviewee and a coworker might target the interviewee’s trait “agreeableness”. ATIC is measured by presenting the interviewee with the questions after finishing the interview. The interviewee then responds in free text what they think was measured (for an example, see Melchers et al. 2020a). Although ATIC research greatly advanced research on faking, the assessment of ATIC lacks objectivity because ATIC performance is evaluated by human raters (typically rated by independent raters that are not interviewers), and not by veridical, objective response standards. This introduces a similar duality issue as introduced in our deception example above: the assessment of ATIC relies on the raters’ judging abilities.

Although there is no direct social interaction when assessing faking behavior in questionnaires, the issue of objectivity can be solved. Here, performance can be evaluated based on optimal response profiles, i.e., the degree to which pre-defined response profiles are met by participants. Therefore, in this manuscript, the term faking ability refers to the ability to fake self-reports in questionnaires.

To measure a content valid faking ability factor, a diverse set of faking questionnaire tests must be sampled from the universe of faking behavior. First, faking good and faking bad should be represented and within each condition, different instantiations are desirable to allow an abstraction from the specificity of sampled faking behaviors, i.e., the ability to deliver optimal response profiles for faking good in different jobs and the ability to deliver prespecified response profiles for different disorders in faking bad. Second, jobs and disorders should be fakeable; i.e., they should be sufficiently familiar to participants and the responses should be subjective and not verifiable, i.e., faking should not be detectable. Third, faking tests should be in (imaginative) high-stakes situations, e.g., faking for early retirement benefits when faking bad. Fourth, jobs and disorders must allow for generating optimal response vectors, which for jobs can be derived via O*NET NET (Occupational Information Network) ratings (National Center for O*NET Development n.d.) as done in earlier work (Geiger et al. 2018) and for disorders can be based on established diagnostic criteria such as from the DSM-5 (Diagnostic and Statistical Manual of Mental Disorders). For this study, we will follow these criteria when sampling faking ability tests.

### 1.3. Correlates of Faking Ability

Since faking ability is an ability to deceive, we hypothesize that abilities, such as general mental ability and receptive and productive socio-emotional abilities that are related to deception, are also correlated with faking ability. As introduced above, a successful deceiver must avoid cues to deception by aligning the content and their behavior to the deception (Bond and DePaulo 2006, 2008; Vrij 2002). In Table 1, we summarize the components of faking ability which are components of all deception abilities that lead us to hypotheses about relations of faking ability with socio-emotional abilities and general mental abilities.

First, deception requires an understanding of the situational demands (ATIC; Kleinmann et al. 2011) and knowledge of how to meet them. For example, in a typical one-on-one conversation, the deceiver must understand the target’s facial emotion expressions to grasp that the receiver is doubting the lie and use their knowledge to prepare behavioral adjustments to make their deception more believable. Thus, next to general mental ability (Michels et al. 2020; Vrij et al. 2008), abilities necessary to understand social situations (Mueller-Hanson et al. 2006), such as emotion perception and emotion recognition, emotional intelligence, and accumulated knowledge, i.e., crystallized intelligence (gc), should relate to faking ability. With respect to knowledge, it might be argued that only situation-specific knowledge is relevant, but research on the dimensionality of gc hints towards a strong general factor (Schipolowski et al. 2014; Steger et al. 2019), indicating that any gc assessment should correlate with faking ability.

Second, deception requires an adjustment of behavior to the situation. This means automatic emotional reactions that can be cues for deception detection (“leakage” of inconsistent emotional reactions; Porter and ten Brinke 2008) must be inhibited and instead, emotional behavior fitting the situation must be expressed. For example, a deceiver might want to convey a sad state but actually feel happy about successfully deceiving others (ten Brinke and Porter 2012). In order to make the deception believable, the happy expression must be inhibited and replaced by a sad expression. Consequently, abilities related to emotional behavior regulation, specifically regulating facial expressions, should relate to deception abilities, including faking ability.

However, because faking ability here is assessed based on faked questionnaires without a direct social interaction, some precautions must be made. Deceiving in a questionnaire requires understanding what the assessment is about, i.e., the ability to identify criteria (ATIC; Kleinmann et al. 2011; König et al. 2006), but without the social interaction, this does not necessarily include emotional perception. Similarly, adjusting behavior in faking questionnaires means producing a deceiving response profile, which is similar to simulating or posing emotions, but does not require regulating facial expressions. Consequently, it might be argued that because the interpersonal aspects requiring socio-emotional abilities are missing in faking ability, general cognitive abilities, such as fluid and specifically crystallized intelligence should have a larger correlation faking ability than with socio-emotional abilities. Specifically, regulation of emotions, such as suppression might correlate the least with faking ability.

While the relationship of faking ability with general mental ability is well-documented (Geiger et al. 2018; MacCann 2013; Pauls and Crost 2005; Pelt et al. 2018; Raymark and Tafero 2009), faking ability is also related to socio-emotional abilities and traits, (emotion perception (Geiger et al. 2018) and trait emotional intelligence (Pelt et al. 2018)), and more importantly, these relations also hold after controlling them for shared variance with general mental ability and still results in medium effect sizes. So, although direct social interaction is missing, the socio-emotional traits determining the success of this social interaction correlate with faking ability. This might be explained by communalities from a general socio-emotional abilities factor: the general ability to navigate social situations, or a commonsense factor of social interaction, which also helps with successful faking. Evidence for such a socio-emotional abilities factor exists (Geiger et al. forthcoming a; Hildebrandt et al. 2015; MacCann et al. 2014; Schlegel et al. 2019; Schlegel and Scherer 2018), but its relation with faking ability is still poorly understood because prior work either only used a single test for emotion recognition (Geiger et al. 2018), or only used a self-report emotional intelligence questionnaire (Pelt et al. 2018) which represents socio-emotional abilities poorly (Olderbak and Wilhelm 2020).

### 1.4. Current Study

In this study, we try to fill the gap of understanding of the relationship between faking ability and socio-emotional abilities. To do this, we follow established standards of psychological testing (Cronbach 1949): we assess both faking ability and the covariates with multiple tests that adhere to standards of maximal performance testing. Specifically, we use existing (faking good) and establish new (faking bad) faking ability tests that allow for veridical response scoring, actually measuring faking ability (as described in Geiger et al. 2018) and not faking extent, as some earlier studies have done (Pelt et al. 2018; Raymark and Tafero 2009). Measuring faking extent means that faked response vectors are compared to honest response vectors, whereas measuring faking ability, as we introduce in detail below, means comparing faked response vectors to optimum profiles.

As covariates, theoretically, tests of emotional intelligence are of interest, but these mostly rely on situational judgment procedures that do not fully meet the standards of aptitude testing (Wilhelm 2005). We, therefore, rely on recent developments in the field by using psychometric ability tests of facial emotion perception, facial emotion posing, and facial pain expression regulation tests. Additionally, considering the general positive manifold of human abilities (Spearman 1904), we include working memory capacity tests as markers of general mental ability (g) and control relations for individual differences in g.

The first goal of the present study is to extend the measurement of faking ability by including faking bad indicators and to compare competing measurement models for this broader ability. We present a new measurement instrument to assess individual differences in the ability to fake bad and administer it jointly with established measures of faking good ability (Geiger et al. 2018). This approach allows us to compare measurement models and thus test whether the ability to fake good and bad is a homogeneous construct, or if these are rather represented by distinct abilities.

The second goal of the present study is to embed faking ability into a nomological net of other socio-emotional abilities. We will correlate faking ability with receptive and productive socio-emotional ability factors and general cognitive ability. We expect faking ability to correlate moderately with the ability to simulate and the ability to perceive facial expressions of emotion. Furthermore, because we foremost consider faking ability a socio-emotional ability, we expect it to have a smaller correlation with working memory capacity. However, some prior work also hints towards equal or larger correlations with markers of general mental abilities. Finally, we expect faking ability to have the smallest correlation with the ability to suppress facial expressions of emotion. To ensure that communality among faking ability and socio-emotional abilities is not merely due to generalized positive manifold, we additionally test these correlations after controlling the socio-emotional abilities for general mental ability, assessed with working memory capacity tests, the best indicators of g (Kyllonen and Christal 1990; Wilhelm et al. 2013).

## 2. Methods & Materials

### 2.1. Sample

Data from this study has been used in another publication (Geiger et al. forthcoming b), but the faking ability data has not been published elsewhere. To recruit a relatively diverse sample, we used a variety of channels, such as radio ads, flyers, posters, snowball sampling, and direct contact on shopping streets. Reimbursement for the study (125€) was substantial because the 134 participants received pain stimulation (Geiger et al. forthcoming b). Sex (49% female) was essentially balanced and ages ranged from 18 to 50 years (*AM* = 32.95; *SD* = 9.61). The study was approved by the local university’s ethics committee.

### 2.2. Procedure

The study consisted of two parts. Part one was conducted in a physiological laboratory, included the pain regulation test, and took about 4 h due to an extensive pain stimulation design (described in Gruss et al. 2019). Here, we will only consider the pain regulation test from part one. Part two took place in a computer laboratory. Participants completed all other measures in approximately 2 h. Some measures from part two are not included in the present paper or only in the Appendix A (that is, self-report measures of personality, alexithymia, emotion-specific empathy, and emotion regulation, as well as measures of mental speed). Most participants completed both parts on different days with up to one week in between testing sessions but, upon request, some participants completed both parts on the same day.

### 2.3. Materials

#### 2.3.1. Faking Ability Measures

**Faking Bad.** In developing faking bad tests, we followed the four criteria for sampling faking ability tests introduced earlier. Faking bad typically occurs in clinical settings (Hall and Hall 2011). Therefore, we designed two faking bad ability tests in this context. Participants were asked to imagine a situation where they fake a psychological health questionnaire during a doctor’s appointment in order to receive social welfare benefits. In the first test, participants were asked to malinger a major depression (faking bad depression test) to gain extended sick leave and funding for recovery at a health resort. In the second test, they were asked to malinger a somatization disorder (faking bad somatization test) to gain early retirement funding. We chose these two psychological disorders because they are well-known due to relatively high lifetime prevalence, cannot be diagnosed based on physiological data, and are amenable to drug-free treatment (because medication and its side effects are highly likely to be considered aversive).

**Psychological Health Questionnaire**. Participants were asked to malinger in a questionnaire composed from DSM-5 (Diagnostic and Statistical Manual of Mental Disorders) Cross-Cutting Symptom Measures (DSM5-CCSM; American Psychiatric Association 2013). This is a collection of self-report questionnaires for patient evaluation. The evaluation procedure consists of a level 1 questionnaire with one to three questions per disorder domain (e.g., two items regarding depression). The questions are answered on a 5-pt rating scale from 0 to 4. For most disorder domains, having any level 1 items of a domain answered with a 2 or higher indicates that further investigation in this domain is required. If so, participants are asked to answer the level 2 questionnaire for the respective disorder. Level 2 questionnaires ask additional and more detailed questions about the disorder and are used to evaluate the severity of a disorder in four categories: none to slight, mild, moderate, or severe.

To construct a psychological health questionnaire, we used the items of the depression and somatization scales from both levels of the DSM5-CCSM. As we deemed it too easy to distinguish depression from somatization items when only these are presented in a questionnaire, we added the items from the anxiety and anger disorder scales as additional distractors. Level 1 and 2 items were administered jointly in the same questionnaire. Some level 1 items are redundant to level 2 items. For example, the level 1 depression item “feeling down, depressed, or hopeless” is redundant to the items “I feel hopeless”, “I feel depressed”, and “I felt sad” from the level 2 questionnaire. Thus, we only kept three level 1 items (one each for depression, somatization, and anxiety), totaling 37 items. The items of the psychological health questionnaire are presented in Table 2. Items were presented in different pre-randomized orders for every condition the questionnaire was used in.

In order to standardize the different rating scales of the combined DSM-5 level 2 questionnaires, we had participants use the 5-pt rating scale from the Brief Symptom Inventory (Derogatis and Spencer 1993), ranging from “never” (1) to “always” (5). Questions always referred to how one felt during the last seven days. In addition to faking this questionnaire twice, participants also answered this questionnaire honestly as the very first questionnaire in the second part of the study. We used these honest responses to calculate comorbidity penalties to the faking bad scores (see below).

**Faking Bad Instructions**. The faking bad tests started by asking participants to imagine a situation in which they would like to receive unwarranted social welfare benefits, such as early retirement. Furthermore, they were asked to imagine that they planned on faking a psychological target disorder (depression in one test and somatization disorder in the other test) and now must answer a health questionnaire at a doctor’s appointment accordingly. Additionally, participants received a short prompt about the psychological disorder, such as “On the internet, you read that depression is a psychiatric disorder that is characterized by a depressed mood and avolition”.

Following standards of maximal performance measurement (Cronbach 1949), participants were instructed to give their best. They were explicitly instructed to answer the questionnaire in a way that only supported the presence of the target disorder, but not the other diagnoses, i.e., to avoid comorbid diagnoses. Lastly, they were reminded that it is their explicit task to fake on the questionnaire and not to answer honestly. The complete instructions are reported in the supplemental material.

**Faking Bad Ability Scoring**. The goal of the faking bad tests was to acquire the diagnosis of an intense psychological target disorder, but not of any other comorbid disorder. Thus, participants’ responses could be scored as veridical scores according to what extent they reached this goal. We followed the original questionnaires’ scoring logic to evaluate the diagnoses. Participants received points for achieving the right diagnosis and related additional symptoms and penalties for achieving comorbid diagnoses. These points and penalties were summed to build a faking bad ability score. The independent scoring approaches for both tests (faking bad depression and faking bad somatization) and all summands of the scoring functions are summarized in the supplementary material.

**Faking Bad Summary**. We developed two faking bad tasks in the context of malingering in clinical settings: faking bad depression and faking bad somatization. Participants had to fake one psychological disorder while trying to avoid items from other psychological disorders in a psychological health questionnaire. Ability scores were built according to these goals; i.e., receiving a diagnosis of the target disorder was awarded with points, and receiving a diagnosis of other disorders was penalized. For confirmatory modeling in the results, we used the sum scores of all summands from each faking bad test.

**Faking Good**. Faking good was assessed with three tests instructing participants to fake good in a job assessment context, adhering to the principles of sampling faking ability tests introduced earlier. Participants were asked to complete the Work Style Questionnaire (Borman et al. 1999) for three jobs in fictitious recruitment contexts in a way to maximize their chances of getting hired (Geiger et al. 2018). The three jobs were selected to require distinct personality profiles in order to vary faking good demands across tests. Participants faked applications for security guard (O*NET code: 33-9032.00), insurance policy processing clerk (O*NET code: 43.9041.02), and software developer (O*NET code: 15-1133.00). Faking good ability tests were scored with the profile similarity index shape, which essentially is a correlation between participants’ response vectors and the optimal profile vector. The faking good ability factor had low to acceptable saturation (ω = 0.33–0.58) in earlier work, and demonstrated strong validity (Geiger et al. 2018; Geiger et al. forthcoming a). In confirmatory factor modeling, we used one shape indicator per faking good test.

#### 2.3.2. Covariate Measures

**Facial Emotion Perception**. We administered three tests of facial emotion perception ability from the BeEmo test battery (Wilhelm et al. 2014) to measure receptive socio-emotional abilities. The three tests selected were “identification of emotion expressions from composite faces” (composite emotions, CE), “identification of emotion expressions of different intensity from upright and inverted dynamic face stimuli”, (upright-inverted, UI) and “visual search for faces with corresponding emotion expressions of different intensity” (visual search, VS). These tests are well-established and reliable (ω_CE_ = 0.81; ω_UI_ = 0.62; ω_VS_ = 0.86, Wilhelm et al. 2014)

In the composite emotions test, participants labeled the upper or lower face half of composite faces which expressed different emotional expressions in their upper and lower halves. In the upright-inverted test, participants labeled the emotional expression of short video clips of faces moving from a neutral to an emotional expression (with varying intensity). In the visual search test, participants saw a 3 × 3 face matrix with different emotion expressions of varying intensity. One emotion was always the majority (minimum of five) and participants had to mark the odd-men-out. Following the recommendations from the original authors, the composite emotions and visual search tests were scored with unbiased hit-rates (Wagner 1993). The visual search test was scored as percent correct hits per 3 × 3 matrix. We used one aggregate score per test as indicators in confirmatory factor models.

**Facial Emotion Expression Posing**. The productive ability to pose facial emotion expressions was assessed with a production test and an imitation test. In the production test, participants read an emotional word and were asked to pose this emotion with their facial expressions. In the imitation test, participants were presented with a picture of a face expressing an emotion and had to imitate the expression. Both tests were composed of items based on the six basic emotions (anger, disgust, fear, happiness, sadness, and surprise; Ekman 1992), pain, and neutral trials. In the production test, each condition was presented twice, resulting in 16 items. In the imitation test, each condition was presented four times with different facial identities respectively (two females and two males in each condition), resulting in 32 items. Emotional and neutral faces for the imitation task were drawn from the Berlin Faces Database which was also used to construct the emotion perception tests (Wilhelm et al. 2014). Pain faces were drawn from the STOIC database (Roy et al. 2009).

Participants’ facial expressions were recorded with a video camera for three seconds after a preparation period of seven seconds per item with a resolution of 25 frames per second. Performance in the posing tests was scored with objective facial emotion recognition software; we followed the scoring approach validated in prior work (Geiger et al. forthcoming a; Olderbak et al. 2014). That is, the maximal value of the target expression of a trial, e.g., the highest anger value of all anger values in an anger trial, was extracted as a score. Then, participants’ facial emotion expression scores were controlled for their respective baseline expressions of that emotion assessed in the neutral items of the tests. This process is thoroughly explained in Olderbak et al. (2014).

A general factor of emotion posing ability is reliable with ω = 0.64–0.72 (Geiger et al. forthcoming a). Measurement models of these tests were estimated according to the original paper, i.e., based on emotion parcels (average performance across same emotion trials (e.g., anger) of a test (e.g., imitation)) for production and imitation separately (14 parcels) with a general factor, an imitation specific nested factor, and correlated residuals of same emotion parcels (Geiger et al. forthcoming a).

**Facial Pain Expression Regulation**. The productive ability to regulate facial expressions of pain was assessed with a test design introduced by (Geiger et al. forthcoming b). In this test, participants regulated their facial expressions while experiencing pain. Additionally, they showed genuine pain expressions for baseline assessment. Each item was preceded by a ten second time window during which the following trial was announced and participants prepared and five second expression time during which participants (except for the posing condition) experienced five seconds of pain individually adjusted to their tolerance threshold and had to follow the task at hand, that is, enhance, pose, neutralize, or mask a painful facial expression (with a different emotional expression). Individual tolerance thresholds were measured with a psychophysical pseudo-staircase, upward only threshold estimation task before the expression regulation test. Overall, the test consisted of eight conditions: genuine expression, enhancement, posing, neutralization, masking with happiness, masking with disgust, masking with fear, and masking with surprise. Each condition was presented twice, once with pain stimulation of 90% of the tolerance threshold and once with stimulation at 75%. Thus, the test consisted of 16 items.

Facial expressions were videotaped during the expression time with a time resolution of 25 frames per second. Objective facial expression coding software was used to score average Action Unit activities across a trial. These scores were then used to calculate pain or masking scores. In simulation trials (enhancement, posing) the goal was to achieve high pain scores. In the suppression trials (neutralization, masking), low pain/masking scores were to be achieved by the participants. The test was best modeled as a bifactor model with a general pain expression factor, controlling for baseline pain expressions, and two nested, correlated ability factors: simulation (posing and enhancing) and suppression (neutralizing and masking) with item level indicators. The ability factors have been demonstrated to be reliable (ω_simulation_ = 0.86/0.87; ω_suppression_ = 0.59/0.36) and valid (Geiger et al. forthcoming b).

**General Cognitive Ability**. We assessed working memory capacity as a proxy to general cognitive ability with three tests from a working memory capacity test battery (Wilhelm et al. 2013). To minimize test or stimulus effects, we sampled three tests from different stimulus domains: (1) a letter-color binding (LC-B) test (ω = 0.70) in which participants learned letter-color pairs and had to recall them immediately after learning the last pair of a set; (2) a figural updating (F-U) test (ω = 0.72) in which participants had to remember the last position of colored squares in a frequently updating 3 × 3 grid; (3) a numerical 1-back (N-1b) test (ω = 0.94) in which participants saw one to three boxes with numbers and had to constantly type in the last number in a box when the box was updated to show a new number.

### 2.4. Statistical Analyses

Processing and analyses of data were conducted in R (version 4.0.0, R Core Team 2020) with the packages psych (version 1.9.12; Revelle 2019), lavaan (version 0.6-6; Rosseel 2012), and semTools (version 0.5-3; Jorgensen et al. 2020). Factors in confirmatory factor analyses were identified by fixing factor variances to 1. We tested parameters in factor analyses with a likelihood ratio test (Gonzalez and Griffin 2001) and adjusted χ^2^-distribution (Stoel et al. 2006). Models are deemed acceptable with *CFI (Comparative Fit Index) and TLI (Tucker-Lewis Index) ≥ 0.90, RMSEA (Root Mean Square Error of Approximation) < 0.08 and SRMR (Standardized Root Mean Square Re-sidual) < 0.11* and deemed good with *CFI* and *TLI* ≥ 0.95, *RMSEA* < 0.05 and *SRMR* < 0.08 (Bentler 1990; Hu and Bentler 1999; Steiger 1990).

## 3. Results

All analyses are summarized in R markdown files in the supplemental material on OSF (https://osf.io/3h8j9/?view_only=510fcdb860964f1bab3d08c410456082 (accessed on 3 March 2021)). There and in the Appendix A of this manuscript, we also report additional results not the focus of this manuscript, such as manifest correlations of variables in focus (Table A1), correlations with typical socio-emotional traits, i.e., empathy, emotion regulation, and alexithymia (Table A2), and sex differences in faking ability (Table A3).

### 3.1. Homogeneity of Faking Ability

**Figure 1:**
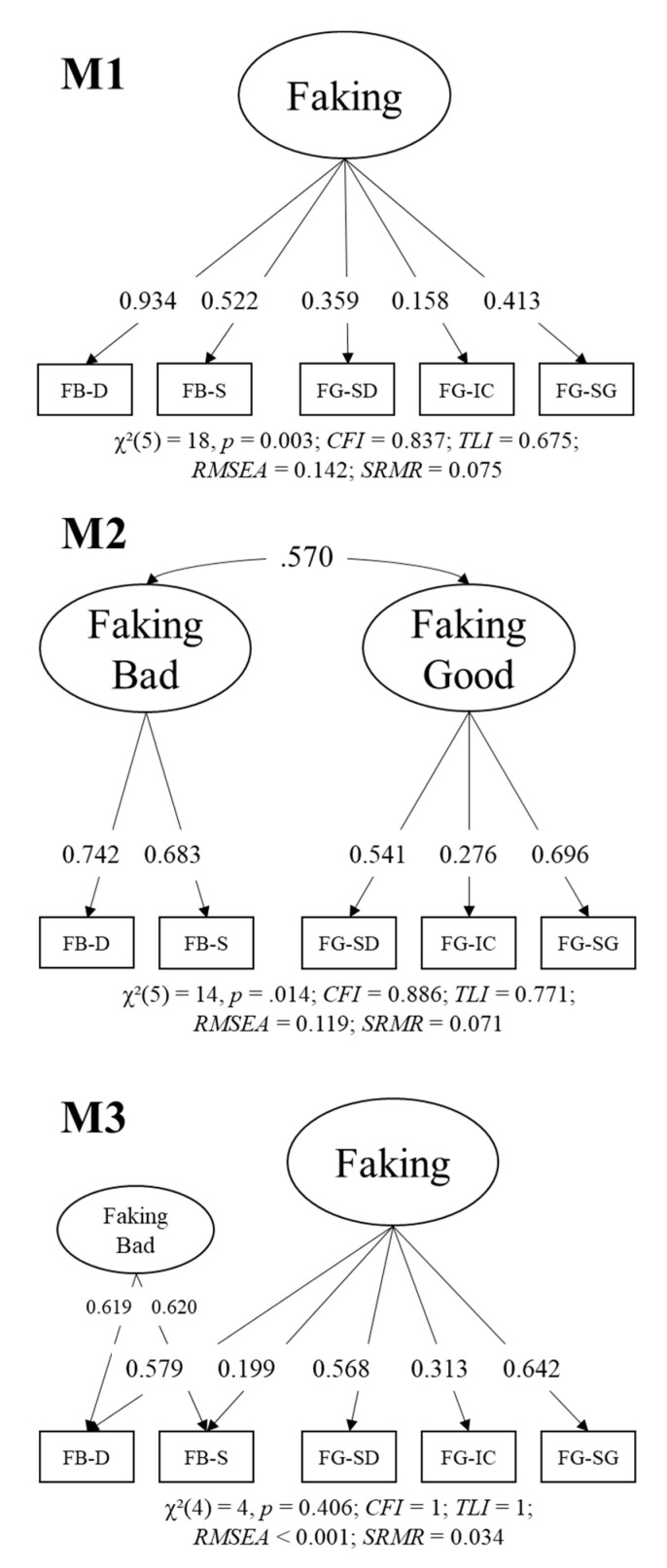
Measurement models of faking ability. FB = faking bad; FG = faking good, D = depression, S = somatization; SD = software developer; IC = insurance policy processing clerk; SG = security guard. Loadings are standardized values.

To test whether faking ability is a homogeneous construct or whether distinct faking good and bad abilities exist, we compared the three measurement models depicted in Figure 1. First, we fitted a general factor model (M1) with a faking ability factor loading on all five faking indicators. The model did not reach acceptable fit: χ^2^(5) = 18, *p* = 0.003; *CFI* = 0.837; *TLI* = 0.675; *RMSEA* = 0.142; *SRMR* = 0.075. Second, we tested a correlated-factors model (M2) with a faking bad and a faking good factor (M2). The two (unstandardized) loadings of the faking bad factors were fixed to equality for local identification. The factors had a large correlation (*r* = 0.570), but model fit was not acceptable either: χ^2^(5) = 14, *p* = 0.014; *CFI* = 0.886; *TLI* = 0.771; *RMSEA* = 0.119; *SRMR* = 0.071. Therefore, we tested a third model: a bifactor model with a general faking ability factor and a nested faking bad factor (M3). Again, the two (unstandardized) loadings of the faking bad factor were fixed to equality for local identification. This model fit very well with χ^2^(4) = 4, *p* = 0.406; *CFI* = 1; *TLI* = 1; *RMSEA* < 0.001; *SRMR* = 0.034. Both factors reached satisfactory saturation given the measurement approach (ω_Faking_ = 0.539; ω_FakingBad_ = 0.641), but it must be noted that loadings on the general faking ability factor varied substantially, with small to medium loadings on faking bad somatization (λ = 0.199) and faking good insurance clerk (λ = 0.313). The other loadings were strong (λ = 0.568–0.642).

We conclude that a general faking ability factor fits the data well as long as specific variation in the faking bad tests is considered as well. The specific faking bad factor could either represent an independent ability or method variance (for details on interpreting specific factors in bifactor models, see e.g., Eid et al. 2008) because the faking bad tests were scored differently than the faking good tests. Convergent relations of both factors will be examined in the next step. The absence of convergent relations of the nested faking bad factor would endorse an interpretation as a method factor. Conversely, substantial convergent relations of this factor would endorse an interpretation as a specific ability trait.

### 3.2. Faking Ability and Socio-Emotional Abilities

**Figure 2:**
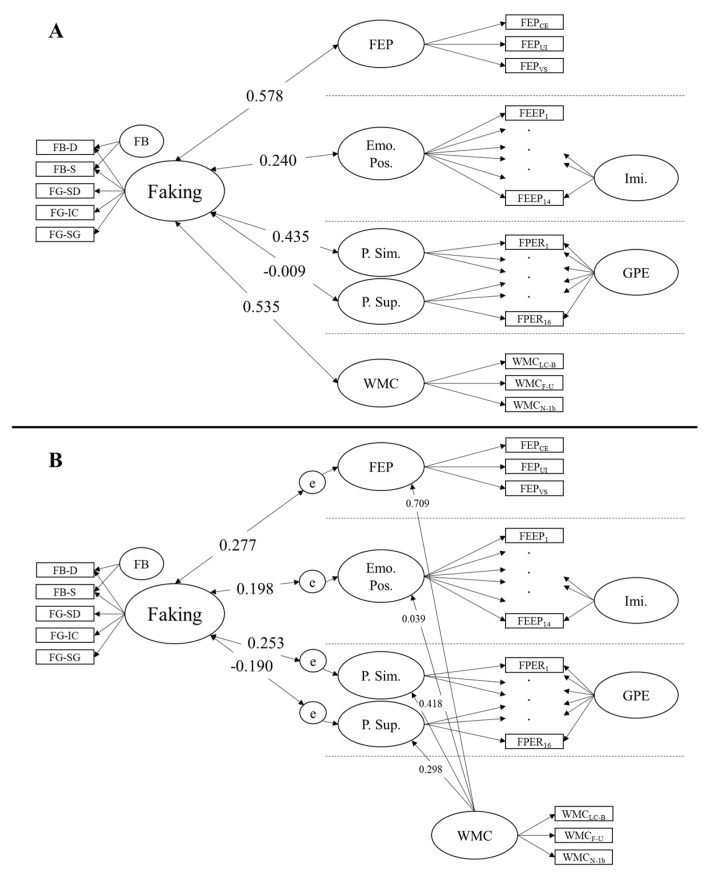
Confirmatory factor analyses to estimate the correlations between faking ability and covariates (schematic measurement models) before (**A**) and after (**B**) controlling for general mental ability. Dashed lines indicate that models were estimated separately per covariate. FB = faking bad; FG = faking good, D = depression, S = somatization; SD = software developer; IC = insurance policy processing clerk; SG = security guard; FEP = facial emotion perception ability; CE = composite emotions; UI = upright-inverted; VS = visual search; Emo. Pos. = emotion posing ability; Imi. = imitation; FEEP = facial emotion expression posing ability; P.Sim = pain simulation; P.Sup. = pain suppression; GPE = general pain expression; FPER = facial pain expression regulation; WMC = working memory capacity; LC-B = letter-color binding; F-U = figural updating; N-1b = numerical 1-back.

Correlations of faking ability with socio-emotional abilities and general mental ability were estimated in separate confirmatory factor analyses. The models and the correlations are summarized in Figure 2A (please note that the correlations with faking bad were estimated but are not displayed because we found no systematic correlations with this factor; see below). To estimate the correlation of faking ability with receptive socio-emotional abilities, we modeled a general factor of facial emotion perception (with the three indicators of the facial emotion perception tests loading on the factor) jointly with the faking ability model (M3) and allowed the factors to correlate. This model had a good fit (χ^2^(17) = 17, *p* = 0.460; *CFI* = 1; *TLI* = 1; *RMSEA* < 0.001; *SRMR* = 0.039). The facial emotion perception factor was reliable with ω = 0.739. Faking ability had a large correlation with facial emotion perception (*r* = 0.578, *p* < 0.001). This correlation was slightly larger than expected, supporting our hypothesis.

Next, we modeled faking ability jointly with facial emotion expression posing. The latter measurement model is depicted in the supplemental material. This measurement model consists of a general facial emotion posing ability factor, a nested imitation factor, and six correlated residuals between the same emotion trials. There was no correlation between the residuals of the pain posing parcels because the pain imitation parcel was exactly identified by the loading on it, thus leaving no residual. The joint model of faking ability and facial emotion posing ability was only partly acceptable (χ^2^(137) = 210, *p* < 0.001; *CFI* = 0.877; *TLI* = 0.846; *RMSEA* = 0.064; *SRMR* = 0.103), which was due to the facial emotion posing ability model. However, because this model has been validated several times in other studies (Geiger et al. forthcoming a), we did not modify it. The general facial emotion expression posing factor had a low reliability of ω = 0.365. We found a small correlation between the faking ability and the general facial emotion posing factors (*r* = 0.240, *p* = 0.059), which is slightly smaller than expected, but in the expected direction.

Pain regulation ability was also modeled according to prior work (Geiger et al. forthcoming b) with correlated specific simulation and suppression ability factors. When modeled jointly with faking ability, the model had acceptable to good fit (χ^2^(165) = 259, *p* < 0.001; *CFI* = 0.943; *TLI* = 0.928; *RMSEA* = 0.066; *SRMR* = 0.063). The simulation factor was very reliable (ω = 0.867), while the reliability of the suppression factor was low (ω = 0.386). As expected, we found a moderate correlation of faking ability with simulation (*r* = 0.435, *p* = 0.001), but, against our expectations, no correlation with suppression (*r* = −0.009, *p* = 0.475).

As indicators of general mental ability, we modeled a general factor explaining the test scores from the three working memory capacity tests. The joint model with faking ability had a very good fit (χ^2^(17) = 12, *p* = 0.772; *CFI* = 1; *TLI* = 1; *RMSEA* < 0.001; *SRMR* = 0.038). The general mental ability factor was reliable with ω = 0.779. Against our expectations but in line with earlier findings, faking ability had a large correlation with general mental ability of *r* = 0.535 (*p* < 0.001).

In the same models, we also tested the correlations of the faking bad factor with these covariates to guide interpretation of this factor as a trait or method factor. We found no systematic correlation of this factor. Correlations were distributed around zero: facial emotion perception: *r* = 0.060 (*p* = 0.337); facial emotion expression posing: *r* = 0.067 (*p* = 0.323); facial pain expression simulation: *r* = −0.146 (*p* = 0.156); facial pain expression suppression: *r* = 0.227 (*p* = 0.057); general mental ability: *r* = 0.187 (*p* = 0.091). This endorses an interpretation of the faking bad factor as a methods factor.

**Specific Socio-Emotional Abilities Relations**. Due to the unexpectedly strong relation between general mental ability and faking ability, we explored whether the correlations of socio-emotional abilities with faking ability were due to shared variance with general mental ability. Therefore, we ran three additional models correlating faking ability with (1) facial emotion perception, (2) facial emotion expression posing, and (3) pain expression simulation and suppression, after controlling these factors for general mental ability. To do so, we added the general mental ability factor to the previously estimated correlation models and allowed a regression of general mental ability (independent variable) on the socio-emotional ability factors (dependent variable), and let the residual correlate with faking ability. These models are summarized in Figure 2B. The general cognitive ability factor had a large effect on facial emotion perception (γ = 0.709), a medium effect on pain expression simulation (γ = 0.418), and a small effect on pain expression suppression (γ = 0.298), but no effect on facial emotion posing (γ = 0.039). All correlations of faking ability with socio-emotional abilities decreased. However, except for suppression, which was zero to begin with, the partial correlations still were small in size (facial emotion perception: *r* = 0.277, *p* = 0.041; facial emotion expression posing: *r* = 0.198, *p* = 0.081; facial pain expression simulation: *r* = 0.253, *p* = 0.033; facial pain expression suppression: *r* = −0.190, *p* = 0.088).

## 4. Discussion

### 4.1. Summary & Interpretation of Results

**Step 1: Homogeneity of Faking Ability**. We introduced a new approach to measure faking ability with two tests of faking bad in a psychological health questionnaire. Additionally, we administered three more established tests of faking good ability in a job assessment context. This allowed us to investigate the question of whether faking good and bad are a homogeneous construct. Obviously, faking behavior differs between faking good and bad (Bensch et al. 2019). However, whether this results in distinct factors of faking ability, an essential determinant of faking performance (Geiger et al. 2018), was a previously unresolved question. A bifactor measurement model with a general factor of faking ability loading on all five faking tests and a nested faking bad factor loading on the two faking bad tests (Figure 1, M3) fit the data best. Based on this model, we can conclude that faking ability can in fact be understood as a homogeneous construct, i.e., an overarching ability to fake determines success in both faking good and bad tests. The factor must be interpreted with caution however, because it only achieved a satisfactory level of saturation and the specific faking bad factor had a slightly higher saturation.

Thus, there was specific variation in the two faking bad tests that could be modeled by a specific and reliable faking bad factor that however did not systematically correlate with any of our covariate abilities. Based on our study design and these findings—as one of our reviewers pointed out—two interpretations of this factor are reasonable: (a) the specific faking bad factor represents a specific faking bad ability possibly related to specific knowledge about disorders that was not covered in our list of covariates or (b) this factor represents methods variation that is due to the different scoring procedures (symptom-based scoring instead of profile similarity metric of shape). Future studies with more faking ability tests varying scoring procedures across faking good and bad conditions and including knowledge tests might solve the question of which explanation is more likely to be correct.

**Step 2: Faking Ability and Socio-Emotional Ability**. The general faking ability factor did show moderate to strong convergent validity with other socio-emotional abilities. We found a large correlation with the receptive ability facial emotion perception, a medium correlation with facial pain expression simulation, and a small correlation with facial emotion expression posing. Furthermore, women performed slightly better at faking, which corresponds to a similar finding in emotion perception (Olderbak et al. 2019b; Thompson and Voyer 2014). Only the ability to suppress facial expressions of pain was unrelated to faking ability. Given that this correlation was expected to be the smallest amongst the convergent relations, we conclude that the overall pattern of correlations endorses the perspective that faking ability can be understood as a socio-emotional ability.

Although we only expected a small correlation of faking ability with working memory capacity as a marker of general mental ability, we found a large correlation that was also stronger than the correlations of faking ability that we found with productive socio-emotional abilities. This stresses the importance of general cognition for faking ability (Geiger et al. 2018; MacCann 2013; Pauls and Crost 2005) and underlines the phenomenon of positive manifold for socio-emotional ability tests. With such a high correlation it might be argued that faking ability is a general mental ability rather than a socio-emotional one. On the other hand, due to positive manifold, other socio-emotional abilities also share major portions of variance with general mental abilities, yet they carry enough specific variation to form factors that might be interpreted as Stratum II socio-emotional abilities factors in models of intelligence (Hildebrandt et al. 2011, 2015). In line with this, emotion perception, pain simulation, and pain suppression had medium to large correlations with working memory capacity.

To test whether faking ability only relates with socio-emotional abilities due to shared variation with general mental abilities (i.e., general positive manifold) or whether faking ability and socio-emotional abilities share specific variance beyond g, we investigated the correlations of faking ability with socio-emotional abilities after controlling for working memory capacity. Although the correlations of faking ability with socio-emotional abilities dropped when controlling the covariates for general mental ability, they were nevertheless small and meaningful, carrying incremental covariation beyond the shared covariation of general mental ability. This result further supports the interpretation that faking ability also fits in the realm of socio-emotional ability.

We conclude that faking ability is indeed best understood as an overarching construct that is related to other psychometrically-supported socio-emotional abilities and general mental ability. Although the deceptive behavior in faking ability tests is very different from, for example, the simulation of facial expressions or the ability to perceive emotional expressions, our results support the idea that these abilities share a common core.

### 4.2. Implications

This result can help us to expand our understanding of human intelligence. Consensual models of intelligence, such as the Cattel–Horn–Carroll (CHC) model, differentiate complex intelligence abilities (Stratum II factors), such as fluid intelligence, crystallized intelligence, mental speed, and several more explained by a general factor of intelligence (McGrew 2009). Socio-emotional abilities have been proposed as additional Stratum II factors and a study by MacCann et al. (2014) using the Mayer–Salovey–Caruso Test of Emotional Intelligence (Mayer et al. 2003) to test this idea. Although this approach was deemed successful, results must be interpreted with caution, because this test of emotional intelligence does not fulfill the criteria of an intelligence test, such as veridicality (Wilhelm 2005). A recent meta-analysis found only small relations of emotional intelligence with fluid and crystallized intelligence and therefore endorses this critique of emotional intelligence empirically (Olderbak et al. 2019a).

Other approaches to embed socio-emotional abilities in models of intelligence used face and emotion perception and recognition tests to demonstrate that these correlated strongly with g, i.e., demonstrate positive manifold, but still carry substantial specific variation unexplained by other cognitive abilities (Hildebrandt et al. 2011, 2015; Schlegel et al. 2019, 2020). This strong evidence for socio-emotional abilities as Stratum II factor was recently supported by a study showing that productive socio-emotional abilities, such as facial emotion expression posing, also fit in this nomological network of socio-emotional abilities and intelligence (Geiger et al. forthcoming a). The present study adds to this evidence by including faking ability amongst such studied socio-emotional abilities. Based on our results, it could be hypothesized that faking ability is a Stratum I ability in the CHC model, loading on a proposed Stratum II socio-emotional abilities factor. Given that earlier studies also found strong relations to gc (Geiger et al. 2018; MacCann 2013), faking ability might also load on the Stratum II factor gc. We hope future studies will test these hypotheses. In summary, this work contributes to the body of research by demonstrating that socio-emotional abilities can be measured according to criteria of ability tests. Learning more about these abilities broadens our understanding of human intelligence by extending consensual models of intelligence.

Furthermore, our results stress the importance of socio-emotional abilities for successful deception, such as faking, beyond general mental abilities (Geiger et al. 2018). This can help us understand why faked self-reports in high-stakes settings still have criterion validity. For example, although personality questionnaires are faked and thereby lose construct validity in job assessment settings (Schmit and Ryan 1993), they are still incremental predictors of job performance over intelligence (Schmidt and Hunter 1998). Although it is widely assumed that faked personality questionnaires still measure personality and that personality has criterion validity in high-stakes settings, an alternative explanation arises: Faked personality questionnaires measure faking ability, which can be understood as a socio-emotional ability. Socio-emotional abilities are rarely used in job assessments, although they predict job performance (Joseph and Newman 2010). Thus, the incremental predictive validity of faked personality questionnaires might actually be the predictive validity of socio-emotional abilities. This calls for an inclusion of socio-emotional abilities in the applied assessment.

Importantly, faking bad ability tests expand our understanding of faking bad in real life, such as malingering, which might inspire interventions and detection methods. In clinical settings, lying or malingering scales (e.g., the Self-Report Symptom Inventory, SRSI, Merten et al. 2016; or the lying scales from the Minnesota Multiphasic Personality Inventory 2, MMPI-2, Hathaway et al. 1989) are a common practice to identify malingering. However, these scales still often lead to bad decisions because either patients actually experience the malingering symptoms or test-takers identify the malingering items and intentionally avoid them (Singh et al. 2007). The latter is presumably driven by faking ability, which could be tested by extending the faking bad test design to these malingering scales.

### 4.3. Limitations

Although our results clearly indicate that faking ability fits in a realm with other socio-emotional abilities, as it shows substantial positive manifold with other socio-emotional abilities beyond the general mental ability positive manifold, these findings should be replicated. Our assessment of general mental ability was restricted to working memory capacity. Although we chose diverse working memory capacity tests as indicators of general mental abilities, our design did not include other cognitive abilities, such as gc. Certainly, knowledge, as a part of gc, plays a crucial role in deception abilities, including faking ability (Geiger et al. 2018; MacCann 2013) and is also somewhat related to other socio-emotional abilities (e.g., Olderbak et al. 2019a), so gc might account for the correlations of faking ability with socio-emotional abilities. However, in Geiger et al. (2018) emotion perception had relations with faking ability incremental to gc, and given that gc only weakly relates with emotion posing (Geiger et al. forthcoming a), similar findings can be expected for productive socio-emotional abilities. Therefore, we intentionally focused on socio-emotional abilities’ relations to faking ability and did not include gc. Nevertheless, future research should capitalize on multivariate study design (as employed here in order to generalize constructs) while adding more cognitive abilities in order to precisely locate faking ability’s position in a broad nomological network of human abilities.

## 5. Conclusions

Whereas faking good and bad are obviously different processes (Bensch et al. 2019), successful faking always requires high levels of the ability to identify criteria (or demands of the situation, König et al. 2006) and general knowledge (Geiger et al. 2018), presumably to the same extent. Thus, it was an open question whether there is a general ability to fake on questionnaires, or whether there are distinct faking good and bad abilities. Our results indicate that faking ability is best understood as a general ability. From prior work, we knew that successful faking requires general mental abilities and knowledge (Geiger et al. 2018; MacCann 2013; Pauls and Crost 2005) and is also related to facial emotion perception (Geiger et al. 2018). In our study, we extended this research by including more socio-emotional abilities and a multivariate test design. We replicated and extended these findings and conclude that faking ability fits in a realm with other socio-emotional abilities.

## Figures and Tables

**Table 1:** Components of faking ability.

Component	Example When Faking	Related Abilities in Other Deception Behavior	Expected *r* Effect Size with Faking Ability
Understanding of the social situation	A malingerer understands, based on the questionnaire composition, which psychopathological questions should be faked.	Emotion perception ability	Medium
		Working memory capacity	Small
Knowledge about the social situation	A job applicant faker knows which personality traits are relevant for a job.	General knowledge	Not included in this study; *r* = 0.50 in (Geiger et al. 2018)
Production of deception behavior	A faker produces faked response vectors deviating from their typical responses.	Emotion posing and simulation	Medium
Suppression of cues to deception	A faker disguises their deception by not faking everything to a maximum.	Emotion suppression	Small

Note: *r* effect sizes refer to weak = 0.10–0.19; small = 0.20–0.29; medium = 0.30–0.49; large ≥ 0.50.

**Table 2:** Psychological health questionnaire.

Code	Item Text	Summand Assignment	
		FB-D	FB-S
D1	I felt worthless.	D_L2D_	S_L2CD_
D2	I felt that I had nothing to look forward to.	D_Core,_ D_L2D_	S_L2CD_
D3	I felt helpless.	D_L2D_	S_L2CD_
D4	I felt sad.	D_Core,_ D_L2D_	S_L2CD_
D5	I felt like a failure.	D_L2D_	S_L2CD_
D6	I felt depressed.	D_Core,_ D_L2D_	S_L2CD_
D7	I felt unhappy.	D_Core,_ D_L2D_	S_L2CD_
D8	I felt hopeless.	D_L2D_	S_L2CD_
S1	I felt stomach pain.	D_L2CS_	S_L2S_
S2	I felt back pain.	D_L2CS_	S_L2S_
S3	I had pain in my arms, legs, or joints (knees, hips, etc.)	D_L2CS_	S_L2S_
S5	I had headaches.	D_L2CS_	S_L2S_
S6	I felt chest pain.	D_L2CS_	S_L2S_
S7	I felt dizzy.	D_L2CS_	S_L2S_
S8	I fainted.	D_L2CS_	S_L2S_
S9	I felt my heart pound or race.	D_L2CS_	S_L2S_
S10	I was short of breath.	D_L2CS_	S_L2S_
S11	I had pain or problems during sexual intercourse.	D_L2CS_	S_L2S_
S12	I had constipation, loose bowels, or diarrhea.	D_L2CS_	S_L2S_
S13	I had nausea or indigestion.	D_L2CS_	S_L2S_
S14	I felt tired or had low energy.	D_Add_	S_L2S_
S15	I had trouble sleeping.	D_Add_	S_L2S_
AG1	I was irritated more than people knew.	D_L2CAG_	S_L2CAG_
AG2	I felt angry.	D_L2CAG_	S_L2CAG_
AG3	I felt like I was ready to explode.	D_L2CAG_	S_L2CAG_
AG4	I was grouchy.	D_L2CAG_	S_L2CAG_
AG5	I felt annoyed.	D_L2CAG_	S_L2CAG_
AX1	I felt fearful.	D_L2CAX_	S_L2S-AX_
AX2	I felt anxious.	D_L2CAX_	S_L2S-AX_
AX3	I felt worried.	D_L2CAX_	S_L2S-AX_
AX4	I found it hard to focus on anything other than my anxiety.	D_L2CAX_	S_L2CAX_
AX5	I felt nervous.	D_L2CAX_	S_L2CAX_
AX6	I felt uneasy.	D_L2CAX_	S_L2CAX_
AX7	I felt tense.	D_L2CAX_	S_L2S-AX_
DL1	I felt little interest or pleasure in doing things.	D_L1D_	S_L1CD_
SL1	I had the feeling that my illnesses were not being taken seriously enough.	D_L1CS_	S_L1S_
AXL1	I avoided situations that made me anxious.	D_L1CAX_	S_L1CAX_

Note: D = depressive disorder; S = somatic Symptom disorder; AG = anger disorder; AX = anxiety disorder; C = comorbid disorder; L1 = level 1; L2 = level 2.

## Supplementary Materials

Data, analyses syntax and results reports are available on OSF (https://osf.io/3h8j9/).

## Appendix A

**Table A1:** Manifest correlations of test/subscale scores.

	(2)	(3)	(4)	(5)	(6)	(7)	(8)	(9)	(10)	(11)	(12)	(13)	(14)	(15)	(16)	(17)	(18)	(19)	(20)	(21)	(22)	(23)	(24)
(1) Faking bad depression	0.50	0.36	0.14	0.33	0.27	0.36	0.22	0.19	0.14	0.20	0.02	0.10	0.24	0.34	0.20	0.04	0.09	0.01	−0.01	−0.15	−0.04	−0.04	−0.09
(2) Faking bad somatization		0.18	−0.08	0.10	0.12	0.11	0.05	0.02	0.01	0.01	0.10	0.06	0.20	0.22	0.19	0.14	0.03	−0.09	0.04	−0.12	0.09	−0.03	−0.01
(3) Faking good security			0.21	0.35	0.23	0.20	0.26	0.12	0.00	0.09	0.13	0.09	0.24	0.28	0.20	−0.18	0.16	0.14	0.03	−0.14	0.03	−0.07	−0.06
(4) Faking good insurance				0.14	0.15	0.11	0.04	0.06	−0.08	0.01	−0.01	0.02	−0.03	0.14	0.13	−0.14	0.11	0.22	−0.14	−0.09	−0.19	−0.04	−0.15
(5) Faking good software					0.31	0.27	0.21	0.18	0.14	0.18	0.18	−0.04	0.18	0.33	0.15	−0.10	0.10	0.01	0.06	−0.08	−0.06	−0.08	−0.09
(6) Emotion perception UI						0.41	0.52	0.30	0.26	0.32	0.20	−0.03	0.33	0.21	0.32	0.01	0.26	0.05	−0.17	−0.08	−0.13	−0.27	−0.18
(7) Emotion perception VS							0.51	0.22	0.34	0.30	−0.07	0.22	0.38	0.45	0.44	0.04	0.24	0.06	−0.11	0.05	−0.06	−0.28	−0.10
(8) Emotion perception EC								0.17	0.17	0.20	−0.02	0.15	0.32	0.35	0.42	−0.07	0.33	0.18	−0.06	−0.04	−0.04	−0.27	−0.12
(9) Emotion posing production									0.58	0.93	0.29	0.10	0.18	0.11	0.06	0.02	0.23	0.00	−0.26	−0.13	−0.16	−0.18	−0.19
(10) Emotion posing imitation										0.83	0.18	0.00	0.13	0.25	0.05	0.04	0.21	−0.11	−0.23	−0.02	−0.02	−0.10	−0.05
(11) Emotion posing total											0.27	0.07	0.18	0.18	0.07	0.03	0.25	−0.05	−0.27	−0.10	−0.11	−0.17	−0.15
(12) Pain regulation simulation												−0.45	0.05	0.10	−0.01	0.01	0.03	0.03	−0.11	−0.12	−0.05	−0.05	−0.09
(13) Pain regulation suppression													0.22	0.26	0.03	−0.13	−0.02	−0.06	0.07	0.12	0.15	−0.01	0.12
(14) WMC binding														0.54	0.49	−0.05	0.16	0.00	−0.16	−0.18	−0.05	−0.23	−0.17
(15) WMC updating															0.52	−0.02	0.05	−0.02	0.00	−0.01	0.09	−0.18	−0.02
(16) WMC 1back																−0.09	0.14	−0.04	0.09	−0.07	−0.01	−0.15	−0.08
(17) ESE affective empathy																	0.25	0.16	−0.08	0.20	0.01	0.02	0.09
(18) ESE cognitive empathy																		0.20	−0.30	−0.18	−0.38	−0.38	−0.38
(19) ERQ reappraisal																			−0.16	−0.13	−0.16	−0.20	−0.19
(20) ERQ suppression																				0.28	0.60	0.32	0.52
(21) Alexithymia DIF																					0.52	0.43	0.80
(22) Alexithymia DDF																						0.51	0.88
(23) Alexithymia EOT																							0.74
(24) Alexithymia total																							

Note: UI = upright-inverted; VS = visual search; EC = emotion composite; WMC = working memory capacity; ESE = emotion-specific empathy; ERQ = emotion regulation questionnaire; DIF = difficulties identifying feelings; DDF = difficulties describing feelings; EOT = externally oriented thinking.

**Table A2:** Correlations with typical socio-emotional traits.

Covariate		Faking Ability		Faking Bad	
	ω	*r*	*p*	*r*	*p*
Emotion-Specific Empathy	1	0.208	0.278	0.088	0.500
Emotion Regulation Questionnaire Reappraisal	0.705	0.288	0.024	−0.129	0.180
Emotion Regulation Questionnaire Suppression	0.709	0.084	0.276	−0.036	0.394
Alexithymia	0.754	−0.195	0.086	0.024	0.431

Note: Measurement models and fit for covariate constructs are reported and displayed in the supplemental material. Correlations are estimated between latent factors (disattenuated).

**Table A3:** Sex differences in faking ability.

Manifest Level					Latent Level
Faking Test	Females’ (0) AM (SD)	Males’ (1) AM (SD)	Cohen’s *d* (*p*)		Fully Standardized Regression Weight of Sex Predicting Faking Ability
FG Security	0.48 (0.21)	0.42 (0.22)	−0.24 (0.17)	Faking ability factor	β = −0.245 (*p* = 0.035)

Note: *p*-values for Cohen’s *d* effect sizes were estimated via independent sample *t*-tests. Cohen’s *d*s are estimated based on females minus males, so negative values indicate higher abilities in females. Similarly, negative values in the standardized regression weight from the structural equations model indicate higher abilities in females.

## References

[B1-jintelligence-09-00013] American Psychiatric Association (2013). Diagnostic and Statistical Manual of Mental Disorders.

[B2-jintelligence-09-00013] Bensch Doreen, Maaß Ulrike, Greiff Samuel, Horstmann Kai Tobias, Ziegler Matthias (2019). The nature of faking: A homogeneous and predictable construct?. Psychological Assessment.

[B3-jintelligence-09-00013] Bentler Peter M. (1990). Comparative fit indexes in structural models. Psychological Bulletin.

[B4-jintelligence-09-00013] Birkeland Scott A., Manson Todd M., Kisamore Jennifer L., Brannick Michael T., Smith Mark A. (2006). A meta-analytic investigation of job applicant faking on personality measures: Job applicant faking on personality measures. International Journal of Selection and Assessment.

[B5-jintelligence-09-00013] Bond Charles F., DePaulo Bella M. (2006). Accuracy of Deception Judgments. Personality and Social Psychology Review.

[B6-jintelligence-09-00013] Bond Charles F., DePaulo Bella M. (2008). Individual differences in judging deception: Accuracy and bias. Psychological Bulletin.

[B7-jintelligence-09-00013] Borman Walter C., Kubisiak U. Christean, Schneider Robert J., Peterson N., Mumford M., Borman W., Jeanneret R., Fleishman E. (1999). Work styles. An Occupational Information System for the 2st Century: The Development of O*Net.

[B8-jintelligence-09-00013] Côté Stéphane, Gyurak Anett, Levenson Robert W. (2010). The ability to regulate emotion is associated with greater well-being, income, and socioeconomic status. Emotion.

[B9-jintelligence-09-00013] Cronbach Lee J. (1949). Essentials of Psychological Testing.

[B10-jintelligence-09-00013] Derogatis L. R., Spencer P. M. (1993). Brief Symptom Inventory: BSI.

[B11-jintelligence-09-00013] Eid Michael, Nussbeck Fridtjof W., Geiser Christian, Cole David A., Gollwitzer Mario, Lischetzke Tanja (2008). Structural equation modeling of multitrait-multimethod data: Different models for different types of methods. Psychological Methods.

[B12-jintelligence-09-00013] Ekman Paul (1992). An argument for basic emotions. Cognition and Emotion.

[B13-jintelligence-09-00013] Geiger Mattis, Olderbak Sally, Sauter Ramona, Wilhelm Oliver (2018). The “g” in Faking: Doublethink the Validity of Personality Self-Report Measures for Applicant Selection. Frontiers in Psychology.

[B14-jintelligence-09-00013] Geiger Mattis, Olderbak Sally, Wilhelm Oliver “Show Me What You Got”: The Nomological Network of the Ability to Pose Facial Emotion Expressions.

[B15-jintelligence-09-00013] Geiger Mattis, Hrycyk Lianna, Wilhelm Oliver Hide the Pain, Harold: Individual Differences in the Ability to Regulate Facial Expressions.

[B16-jintelligence-09-00013] Gonzalez Richard, Griffin Dale (2001). Testing parameters in structural equation modeling: Every “one” matters. Psychological Methods.

[B17-jintelligence-09-00013] Gruss Sascha, Geiger Mattis, Werner Philipp, Wilhelm Oliver, Traue Harald C., Al-Hamadi Ayoub, Walter Steffen (2019). Multi-Modal Signals for Analyzing Pain Responses to Thermal and Electrical Stimuli. Journal of Visualized Experiments.

[B18-jintelligence-09-00013] Hall Ryan C. W., Hall Richard C. W., Ziegler M., MacCann C., Roberts R. D. (2011). Plaintiffs Who Malinger: Impact of Litigation on Fake Testimony. New Perspectives on Faking in Personality Assessment.

[B19-jintelligence-09-00013] Hathaway Starke Rosecrans, McKinley John Charnley (1989). MMPI-2: Minnesota Multiphasic Personality Inventory-2: Manual for Administration and Scoring.

[B20-jintelligence-09-00013] Hildebrandt Andrea, Wilhelm Oliver, Schmiedek Florian, Herzmann Grit, Sommer Werner (2011). On the specificity of face cognition compared with general cognitive functioning across adult age. Psychology and Aging.

[B21-jintelligence-09-00013] Hildebrandt Andrea, Sommer Werner, Schacht Annekathrin, Wilhelm Oliver (2015). Perceiving and remembering emotional facial expressions—A basic facet of emotional intelligence. Intelligence.

[B22-jintelligence-09-00013] Hu Litze, Bentler Peter M. (1999). Cutoff criteria for fit indeces in covariance structure analysis: Conventional criteria versus new alternatives. Structural Equation Modeling: A Multidisciplinary Journal.

[B23-jintelligence-09-00013] Jorgensen Terrence D., Pornprasertmanit Sunthud, Schoemann Alexander M., Rosseel Yves (2020). semTools: Useful Tools for Structural Equation Modeling (0.5-3). https://CRAN.R-project.org/package=semTools.

[B24-jintelligence-09-00013] Joseph Dana L., Newman Daniel A. (2010). Emotional intelligence: An integrative meta-analysis and cascading model. Journal of Applied Psychology.

[B25-jintelligence-09-00013] Kleinmann Martin, Ingold Pia V., Lievens Filip, Jansen Anne, Melchers Klaus G., König Cornelius J. (2011). A different look at why selection procedures work: The role of candidates’ ability to identify criteria. Organizational Psychology Review.

[B26-jintelligence-09-00013] König Cornelius J., Melchers Klaus G., Kleinmann Martin, Richter Gerald M., Klehe Ute-Christine (2006). The relationship between the ability to identify evaluation criteria and integrity test scores. Psychology Science.

[B27-jintelligence-09-00013] Kyllonen Patrick C., Christal Raymond E. (1990). Reasoning ability is (little more than) working-memory capacity?. Intelligence.

[B28-jintelligence-09-00013] Law Marvin K. H., Jackson Simon A., Aidman Eugene, Geiger Mattis, Olderbak Sally, Kleitman Sabina (2018). It’s the deceiver, not the receiver: No individual differences when detecting deception in a foreign and a native language. PLoS ONE.

[B29-jintelligence-09-00013] MacCann Carolyn (2013). Instructed faking of the HEXACO reduces facet reliability and involves more Gc than Gf. Personality and Individual Differences.

[B30-jintelligence-09-00013] MacCann Carolyn, Joseph Dana L., Newman Daniel A., Roberts Richard D. (2014). Emotional intelligence is a second-stratum factor of intelligence: Evidence from hierarchical and bifactor models. Emotion.

[B31-jintelligence-09-00013] MacCann Carolyn, Jiang Yixin, Brown Luke ER, Double Kit S., Bucich Micaela, Minbashian Amirali (2020). Emotional intelligence predicts academic performance: A meta-analysis. Psychological Bulletin.

[B32-jintelligence-09-00013] Mayer John D., Salovey Peter, Caruso David R., Sitarenios Gill (2003). Measuring emotional intelligence with the MSCEIT V2.0. Emotion.

[B33-jintelligence-09-00013] McGrew Kevin S. (2009). CHC theory and the human cognitive abilities project: Standing on the shoulders of the giants of psychometric intelligence research. Intelligence.

[B34-jintelligence-09-00013] Melchers Klaus G., Bill Benedikt, Buehl Anne-Kathrin, Rybczynski Katrin, Kühnel Jana (2020a). Identification of the targeted performance dimensions and self-promotion in interviews: Investigations of uncharted waters. European Journal of Work and Organizational Psychology.

[B35-jintelligence-09-00013] Melchers Klaus G., Roulin Nicolas, Buehl AnneKathrin (2020b). A review of applicant faking in selection interviews. International Journal of Selection and Assessment.

[B36-jintelligence-09-00013] Merten Thomas, Merckelbach Harald, Giger Peter, Stevens Andreas (2016). The Self-Report Symptom Inventory (SRSI): A new instrument for the assessment of distorted symptom endorsement. Psychological Injury and Law.

[B37-jintelligence-09-00013] Michels Moritz, Molz Günter, Bermpohl Frederic Maas genannt (2020). The ability to lie and its relations to the dark triad and general intelligence. Personality and Individual Differences.

[B38-jintelligence-09-00013] Mueller-Hanson Rose A., Heggestad Eric D., Thornton G. C. (2006). Individual Differences in Impression Management: An Exploration of the Psychological Processes Underlying Faking. Psychology Science.

[B39-jintelligence-09-00013] National Center for O*NET Development O*NET OnLine. O*NET Resource Center. https://www.onetonline.org/.

[B40-jintelligence-09-00013] Olderbak Sally, Wilhelm Oliver (2020). Overarching Principles for the Organization of Socioemotional Constructs. Current Directions in Psychological Science.

[B41-jintelligence-09-00013] Olderbak Sally, Hildebrandt Andrea, Pinkpank Thomas, Sommer Werner, Wilhelm Oliver (2014). Psychometric challenges and proposed solutions when scoring facial emotion expression codes. Behavior Research Methods.

[B42-jintelligence-09-00013] Olderbak Sally, Semmler Martin, Doebler Philipp (2019a). Four-branch model of ability emotional intelligence with fluid and crystallized intelligence: A meta-analysis of relations. Emotion Review.

[B43-jintelligence-09-00013] Olderbak Sally, Wilhelm Oliver, Hildebrandt Andrea, Quoidbach Jordi (2019b). Sex differences in facial emotion perception ability across the lifespan. Cognition and Emotion.

[B44-jintelligence-09-00013] Pauls Cornelia A., Crost Nicolas W. (2005). Cognitive ability and self-reported efficacy of self-presentation predict faking on personality measures. Journal of Individual Differences.

[B45-jintelligence-09-00013] Pelt Dirk H. M., Linden Dimitri van der, Born Marise Ph (2018). How Emotional Intelligence Might Get You the Job: The Relationship Between Trait Emotional Intelligence and Faking on Personality Tests. Human Performance.

[B46-jintelligence-09-00013] Porter Stephen, Brinke Leanne Ten (2008). Reading Between the Lies: Identifying Concealed and Falsified Emotions in Universal Facial Expressions. Psychological Science.

[B47-jintelligence-09-00013] R Core Team (2020). R: A Language and Environment for Statistical Computing (3.5.2.).

[B48-jintelligence-09-00013] Raymark Patrick H., Tafero Tracey L. (2009). Individual differences in the ability to fake on personality measures. Human Performance.

[B49-jintelligence-09-00013] Revelle William R. (2019). psych: Procedures for Personality and Psychological Research (1.9.12).

[B50-jintelligence-09-00013] Riggio Ronald E., Tucker Joan, Throckmorton Barbara (1987). Social skills and deception ability. Personality and Social Psychology Bulletin.

[B51-jintelligence-09-00013] Rosseel Yves (2012). lavaan: An R Package for Structural Equation Modeling (0.6-6). http://www.jstatsoft.org/v48/i02/.

[B52-jintelligence-09-00013] Roy Sylvain, Roy Cynthia, Éthier-Majcher Catherine, Fortin Isabelle, Belin Pascal, Gosselin Frédéric (2009). STOIC: A Database of Dynamic and Static Faces Expressing Highly Recognizable Emotions.

[B53-jintelligence-09-00013] Schipolowski Stefan, Wilhelm Oliver, Schroeders Ulrich (2014). On the nature of crystallized intelligence: The relationship between verbal ability and factual knowledge. Intelligence.

[B54-jintelligence-09-00013] Schlegel Katja, Scherer Klaus R. (2018). The nomological network of emotion knowledge and emotion understanding in adults: Evidence from two new performance-based tests. Cognition and Emotion.

[B55-jintelligence-09-00013] Schlegel Katja, Fontaine Johnny R. J., Scherer Klaus R. (2019). The Nomological Network of Emotion Recognition Ability: Evidence from the Geneva Emotion Recognition Test. European Journal of Psychological Assessment.

[B56-jintelligence-09-00013] Schlegel Katja, Palese Tristan, Mast Marianne Schmid, Rammsayer Thomas H., Hall Judith A., Murphy Nora A. (2020). A meta-analysis of the relationship between emotion recognition ability and intelligence. Cognition and Emotion.

[B57-jintelligence-09-00013] Schmidt Frank L., Hunter John E. (1998). The Validity and Utility of Selection Methods in Personnel Psychology: Practical and Theoretical Implications of 85 Years of Research Findings. Psychological Bulletin.

[B58-jintelligence-09-00013] Schmit Mark J., Ryan Ann M. (1993). The Big Five in Personnel Selection: Factor Structure in Applicant and Nonapplicant Populations. Journal of Applied Psychology.

[B59-jintelligence-09-00013] Singh Jaspreet, Avasthi Ajit, Grover Sandeep (2007). Malingering of psychiatric disorders: A review. German Journal of Psychiatry.

[B60-jintelligence-09-00013] Sip Kamila E., Lynge Morten, Wallentin Mikkel, McGregor William B., Frith Christopher D., Roepstorff Andreas (2010). The production and detection of deception in an interactive game. Neuropsychologia.

[B61-jintelligence-09-00013] Spearman Charles (1904). “General intelligence” objectively determined and measured. American Journal of Psychology.

[B62-jintelligence-09-00013] Steger Diana, Schroeders Ulrich, Wilhelm Oliver (2019). On the dimensionality of crystallized intelligence: A smartphone-based assessment. Intelligence.

[B63-jintelligence-09-00013] Steiger James H. (1990). Structural model evaluation and modification: An interval estimation approach. Multivariate Behavioral Research.

[B64-jintelligence-09-00013] Stoel Reinoud D., Garre Francisca Galindo, Dolan Conor, Wittenboer Godfried Van Den (2006). On the likelihood ratio test in structural equation modeling when parameters are subject to boundary constraints. Psychological Methods.

[B65-jintelligence-09-00013] Ten Brinke Leanne, Porter Stephen (2012). Cry me a river: Identifying the behavioral consequences of extremely high-stakes interpersonal deception. Law and Human Behavior.

[B66-jintelligence-09-00013] Tett Robert P., Simonet Daniel V. (2011). Faking in Personality Assessment: A “Multisaturation” Perspective on Faking as Performance. Human Performance.

[B67-jintelligence-09-00013] Thompson Ashley E., Voyer Daniel (2014). Sex differences in the ability to recognise non-verbal displays of emotion: A meta-analysis. Cognition and Emotion.

[B68-jintelligence-09-00013] Viswesvaran Chockalingam, Ones Deniz S. (1999). Meta-Analyses of Fakability Estimates: Implications for Personality Measurement. Educational and Psychological Measurement.

[B69-jintelligence-09-00013] Vrij Aldert, Brace N., Westcott H. (2002). Telling and detecting lies. Applying Psychology.

[B70-jintelligence-09-00013] Vrij Aldert, Fisher Ronald, Mann Samantha, Leal Sharon (2008). A cognitive load approach to lie detection. Journal of Investigative Psychology and Offender Profiling.

[B71-jintelligence-09-00013] Wagner Hugh L. (1993). On measuring performance in category judgment studies of nonverbal behavior. Journal of Nonverbal Behavior.

[B72-jintelligence-09-00013] Wilhelm Oliver, Schulze R., Roberts R. D. (2005). Measures of emotional intelligence: Practice and standards. Emotional Intelligence: An International Handbook.

[B73-jintelligence-09-00013] Wilhelm Oliver, Hildebrandt Andrea Hildebrandt, Oberauer Klaus (2013). What is working memory capacity, and how can we measure it?. Frontiers in Psychology.

[B74-jintelligence-09-00013] Wilhelm Oliver, Hildebrandt Andrea, Manske Karsten, Schacht Annekathrin, Sommer Werner (2014). Test battery for measuring the perception and recognition of facial expressions of emotion. Frontiers in Psychology.

